# Surgical Treatment of Idiopathic Enlargement of the Right Atrium

**DOI:** 10.1155/2018/7241309

**Published:** 2018-09-20

**Authors:** Francesca Chiara Surace, Federica Iezzi, Massimo Colaneri, Marco Pozzi

**Affiliations:** Department of Paediatric and Congenital Cardiac Surgery and Cardiology, Azienda Ospedaliero Universitaria, Ospedali Riuniti Ancona “Umberto I-G. M. Lancisi-G. Salesi”, Ancona, Italy

## Abstract

Idiopathic enlargement of the right atrium (IERA) is a very rare abnormality. Approximately one-half (48%) of the patients with a congenital enlargement of the right atrium have no symptoms. When they occur, symptoms include shortness of breath (28% of cases), palpitations (17%), arrhythmias (12%), and in rare cases, right heart failure and extreme tiredness. We report one such case of a young man with a disproportionally enlarged right atrium. The basal transthoracic echocardiogram demonstrated a huge right atrium with a thick smoke pattern and mild tricuspid regurgitation in the absence of congenital heart disease. Magnetic resonance imaging confirmed the right atriomegaly, with initial compression of the right ventricle, and excluded congenital heart defects or absence of pericardium. The patient underwent surgical resection of the right atrial wall and the atriotomy was closed, leaving an atrial chamber of normal consistency and size. The resected atrium had normal and homogeneous wall thickness without significant fibrosis which confirmed the diagnosis of an idiopathic enlargement of the right atrium.

## 1. Introduction

Idiopathic dilation of the right atrium is a congenital anomaly with an unknown pathogenesis. It is defined as an isolated enlargement of the right atrium in the absence of other cardiac lesions known to cause right atrial dilation.

It is difficult to estimate the true incidence of the disease since most patients are asymptomatic, and diagnosis is often made incidentally.

Most of the IERA patients are asymptomatic. However, some patients develop arrhythmias or symptoms of congestive heart failure. Since there have been reports of significant associated symptoms and even sudden death, information about the management of this rare disease is essential in order to make a correct diagnosis and prescribe an appropriate course of treatment.

## 2. Case Report

**Figure 1:**
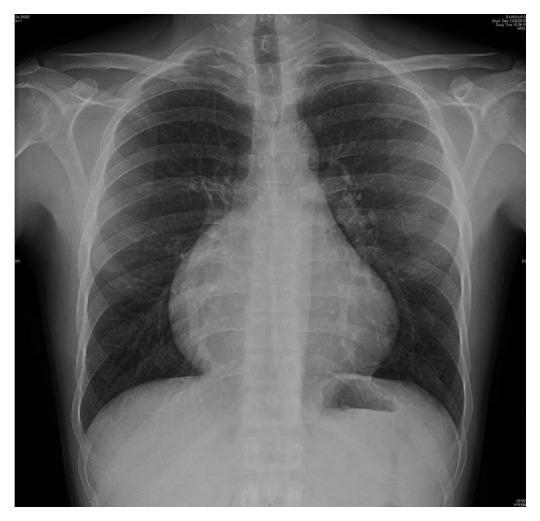
Chest radiograph showing cardiomegaly with right atrial enlargement.

A 23-year-old man (weight 65 kg, height 175 cm, and BSA 1.8 m^2^) with a diagnosis of primitive right atrial enlargement from foetal age was referred to our Centre for cardiological evaluation. Cardiac examination showed increased heart size on percussion and a grade II/VI Levine systolic murmur. No significant pathological findings were found on pulmonary examination. Electrocardiography showed a regular sinus rhythm with a rate of approximately 60 beats/min associated with an abnormal morphology and duration of P wave (enlargement of P wave with duration of 130 msec), together with a low amplitude of QRS complexes in the limb leads. All routine laboratory studies were within normal limits. Chest radiography showed an abnormal cardiac silhouette with increased convexity in the lower half of the right cardiac border and cardiomegaly (Figure 1).

**Figure 2:**
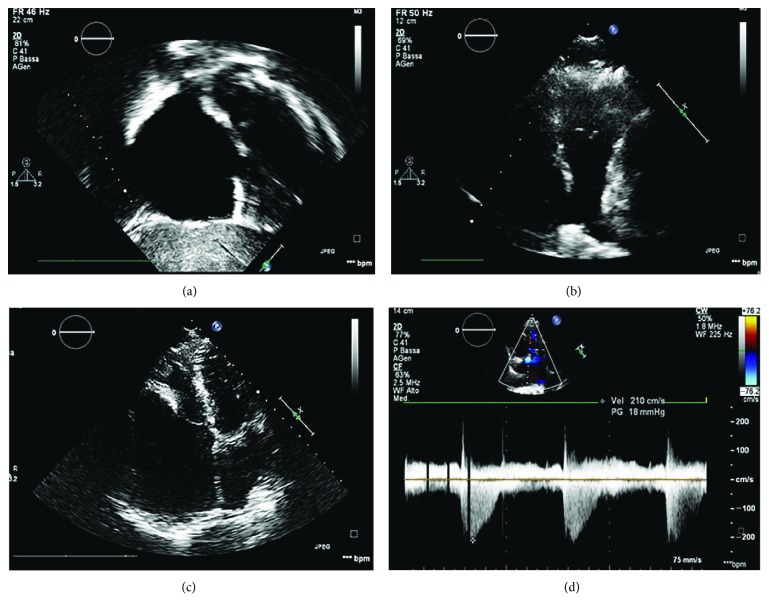
(a) Subxiphoid long-axis view showing abnormal dilation of the right atrium and intact interatrial septum. (b) Modified parasternal short-axis view showing the regular diameter of the pulmonary trunk. (c) Apical 4-chamber view demonstrating the compression of the right ventricle from the right atrium. (d) CW Doppler of tricuspid regurgitation.

Transthoracic two-dimensional echocardiography demonstrated a huge right atrium of about 6.2 cm and a volume of 230 ml/m^2^, with a thick smoke pattern and mild tricuspid regurgitation. The pulmonary arterial pressure was normal (Figure 2). The tricuspid valve was normal without significant annular dilation. No stenosis or abnormal displacement of the tricuspid valve leaflets was detected. No significant regurgitation of the tricuspid valve was found despite a partial distortion of the anterior leaflet and compression of the right ventricle inflow. The right ventricle appeared small and compressed anteriorly by the right atrium (area of RV: 11 cm^2^).

**Figure 3:**
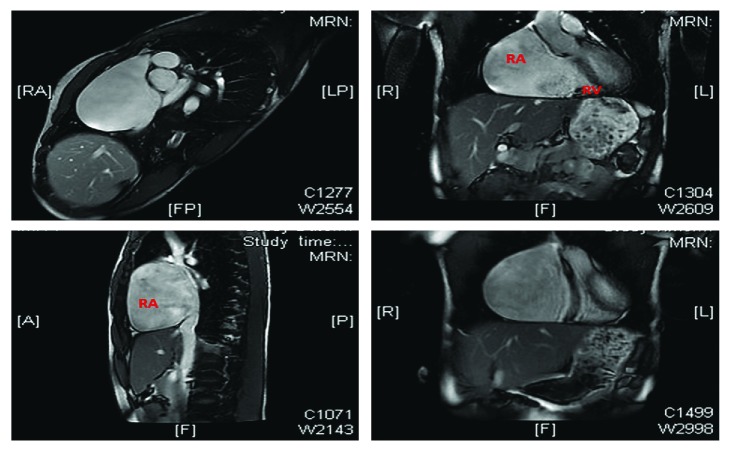
Cardiovascular magnetic resonance (CMR) that confirmed the dilation of the right atrium without congenital heart disease.

Cardiac magnetic resonance imaging showed a marked right atriomegaly (right atrium area: 66.50 cm^2^, volume: 220 ml/m^2^) and normal size of the left atrium (left atrium area: 7.02 cm^2^). The right ventricle was regular in size and global contractility but was partially compressed and dislocated posteriorly, due to the massive enlargement of the right atrium. The left ventricle was regular in dimension, thickness of the wall, and global/segmental contractility (FE VS = 61%). No evident transvalvular jets or areas of late gadolinium enhancement were found. The pericardium was visualized without focal abnormalities or pericardial effusion (Figure 3).

**Figure 4:**
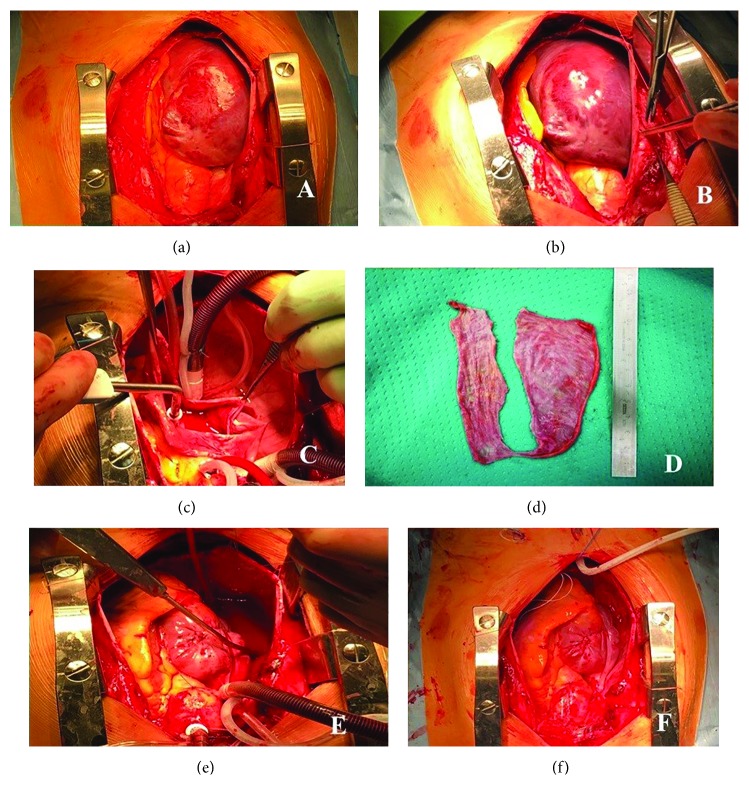
(a, b) Intraoperative view showing the large dilation of the right atrium. (c) The right atrium was fully opened and resected. (d) The resected portion of the right atrium. (e, f) Atrial incision was closed as shown, reconstituting an atrial chamber of approximately normal size and configuration.

Due to the high risk of arrhythmias and thrombus formation in the right atrium, which is a potential risk for pulmonary embolism, the patient underwent cardiac surgery. Through a median sternotomy, cardiopulmonary bypass was established with standard aorta and bicaval cannulation. After the pericardium was opened, the entire anterior surface of the heart was found to be covered with a thin wall in continuity with the right atrium. No atrial appendage as such was apparent. The right atrium was fully opened. The inferior border of the atriotomy was sewn around the anterior part of the tricuspid annulus, and the superior border was brought over the lateral wall of the right atrium as a flap and sewn near the interatrial groove. This provided adequate reduction of the atrial size and reinforcement of the atrial wall (Figure 4).

The histology of the resected atrial wall showed focal hyperplasic areas of smooth muscle cells with polymorphic nuclei surrounded by a few scattered areas of hypertrophic fibrous tissue.

Postoperative transesophageal echocardiogram showed a significant reduction of the right atrium area (23 cm^2^, volume: 93 ml).

The patient was extubated 11 hours after surgery. Complications arose postoperatively with the early appearance of pericardial effusion with leukocytosis and elevated inflammatory markers. This was resistant to conventional medical therapy, which in the end required surgical drainage. Medical therapy of the postpericardiotomy syndrome (ibuprofen 600 mg/TID and colchicine 1 mg/OD) was continued over the subsequent 6 follow-up months without further recurrence of pericardial effusion.

## 3. Discussion

The isolated malformations of the right atrium reported in the literature are very rare cardiac abnormalities, including the following morphological types of disease: diffusely enlarged right atrium (idiopathic enlargement or dilation of the right atrium), single or multiple saccular diverticula of the right atrium, giant right atrial aneurysm (GRAA), right atrial diverticulum (DERA), and aneurysms arising from a part of the right atrium or the coronary sinus [1–3]. The diagnosis of IERA and other right atrial malformations rely on a disproportionately enlarged right atrium compared to the other cardiac chambers in the absence of other cardiac abnormalities.

The differential diagnosis should be made with Ebstein's anomaly, tricuspid or pulmonary stenosis, intracardiac shunts, pulmonary hypertension, severe tricuspid regurgitation, cor triatriatum dexter, pericardial defect (absence of pericardium), and tumors. Idiopathic dilation/enlargement of the right atrium is often an accidental finding during clinical investigation after having identified an enlarged cardiac silhouette on chest X-ray. Instead, patients with diverticula of the coronary sinus usually present supraventricular arrhythmias due to accessory sinoventricular connections [4, 5]. The etiology of all of these abnormalities is unclear.

The first case of a congenital enlargement of the right atrium without any cardiac disease was described by Bailey, while the name “IERA” was first coined by Pastor and Forte in 1961 [6]. Although frequently considered a benign disorder, IERA may be associated with other conditions and morbidity, especially in the adults. Generally, young patients are asymptomatic but sometimes they can be referred for cardiological evaluation due to refractory atrial arrhythmias, heart failure, and thromboembolic complications. There is no consensus about the optimal therapeutic approach. A conservative approach is suggested only in asymptomatic patients with mild to moderate atrial dilation without arrhythmias or signs of compression in adjacent structures. Atrial fibrillation, systemic embolism, and heart failure are the classical complications that can be managed in most of the cases of untreated IERA as well as in GRAA. For this reason, it is reasonable to start low-dose aspirin and regular cardiological follow-up in patients with moderate right atrial dilation, regardless of age [3, 7–9].

Viral etiology of this condition is possible but unlikely because usually foetal viral myocarditis causes global cardiomegaly, heart failure, or arrhythmias and massive pericardial effusion. As already reported by Blondheim et al. [10], two types of idiopathic dilation of the right atrium can be described: one directly connected to a degenerative process of unknown aetiology affecting the atrial myocardium during foetal age and the other, a congenital absence of atrial myocardium and secondary absence of conduction tissue, that is, a true aneurysmal dilation of the right atrium, analogous to Uhl's disease of the right ventricle. The first may have a more benign course, while the second may have associated conduction defects and poor long-term prognosis, including sudden death. Atrial standstill is one such rare association, characterized by the absence of atrial activity on surface and intracavitary electrograms with the absence of atrial mechanical activity.

We report an interesting case of a young man with an isolated severe dilation of the right atrium, causing right ventricle compression without congenital heart disease. The patient was asymptomatic, but the right atrial distention was massive and associated with an initial compression of the right ventricle. The clinical findings led us towards surgical correction to prevent the risk of future thromboembolic complications and dysrhythmias. The most frequent complication of the right atrioplasty technique without pericardial reinforcement is a massive and persistent pericardial effusion caused by a large residual space between the pericardium and the right atrial free wall. Despite what is reported in the literature, no further intervention of pericardiectomy was required in our patient, who responded to the standard medical therapy for postpericardiotomy syndrome.

Even with the aid of modern multi-imaging techniques, the differential diagnosis between IERA and GRAA is very difficult to make. The most consistent data are still provided by histological analysis which documents the presence of thin atrial walls associated with extensive fibrosis in GRAA, while normal wall thicknesses with the regular presence of smooth muscle cells inside is the typical finding in IERA.
